# Immunohistochemistry of advanced glycation end product N^ε^-(carboxymethyl)lysine in coronary arteries in relation to cardiac fibrosis and serum N-terminal-pro basic natriuretic peptide in forensic autopsy cases

**DOI:** 10.1186/s13104-020-05082-6

**Published:** 2020-05-12

**Authors:** Makoto Nogami, Tomoaki Hoshi, Yoko Toukairin, Tomomi Arai, Tadashi Nishio

**Affiliations:** Department of Legal Medicine, Teikyo University School of Medicine, 2-11-1, Kaga, Itabashi-ku, Tokyo, 173-8605 Japan

**Keywords:** Immunohistochemistry, Heart, Advanced glycation end product, N^ε^-(carboxymethyl)lysine, Fibrosis, NT-proBNP, Autopsy

## Abstract

**Objective:**

Advanced glycation end products (AGEs) are known to play important roles in the development of diabetes mellitus and atherosclerosis.　N^ε^-(carboxymethyl)lysine (CML) is the major AGE, and is found in the arterial walls in the heart. The CML involvement in myocardial ischemia has been reported. We studied the immunohistochemical localization of CML in the hearts from forensic autopsies in relation to the age, serum N-terminal-pro basic natriuretic peptide (NT-proBNP), heart weights, and the degree of peri-myocardial fibrous tissues reflecting coronary microvascular infarction and myocardial remodeling.

**Results:**

The CML immunoreactivity in the endothelial cells and intima of arterial walls in the interstitium of ventricular muscles was significantly stronger in the aged group, compatible with the progression of atherosclerosis. The blood level of NT-proBNP, a known useful marker for heart failure, had the positive correlation with the CML immunoreactivity. The degree of fibrosis, heart weights and the histories of hypertension and hyperlipidemia showed positive correlations with the CML immunoreactivity. Our results show the novel positive correlation between the CML immunohistochemistry in the heart vessels and heart conditions, and its future usefulness in the cardiovascular evaluation in histopathology.

## Introduction

Advanced glycation end products (AGEs) are known to accumulate in the state of increased oxidative stress such as diabetes mellitus and aging. It is shown that the major AGE, N^ε^-(carboxymethyl)lysine (CML) in the serum was associated with an increased risk of cardiovascular events in older patients [1]. CML is significantly higher in blood vessels of the left atrial appendage in atrial fibrillation patients as compared to controls, independent of diabetes mellitus [2]. The total amount of CML and left atrial fibrosis in atrial fibrillation and control patients correlated positively [2]. CML was present predominantly on activated endothelium in small intramyocardial blood vessels in patients of acute myocardial infarction (AMI), which might reflect an increased risk for AMI [3]. Previous studies demonstrated the increased accumulation of CML in hearts of diabetes mellitus patients [4, 5].

Myocardial ischemia is caused by a significant coronary stenosis or by coronary microvascular dysfunction. The former involves the whole myocardial ischemia, whereas the latter causes myocardial ischemia localized in small, patchy diffuse areas of myocardium [6]. The diffuse cardiac fibrosis is also observed in the myocardial remodeling in congestive heart failure [7]. The correlation between the diffuse fibrosis in the myocardium resulting from coronary microvascular ischemia or heart failure, and the microvascular CML accumulation is not yet clear, and warrants further study.

In this study, we hypothesized that CML accumulation in the cardiovascular system plays an important role in the development of cardiac ischemia, cardiac failure and fibrosis. Therefore, we evaluated the immunohistochemical presence of CML in hearts of forensic autopsy cases, in relation to the proportions of the interstitial fibrous tissues in the myocardium resulting from microvascular ischemia and myocardial remodeling. Other factors such as heart weights, the age, the clinical presence of hypertension, hyperlipidemia, diabetes mellitus were also studied for the CML immunoreactivity. Since N-terminal-pro basic natriuretic peptide (NT-proBNP) is known to be correlated with heart failure [8] and myocardial fibrosis [9], we also studied the correlation between serum NT-proBNP and CML immunohistochemistry.

## Main text

### Materials and methods

#### Sample collection

The sample collections and analyses were performed as our routine autopsies, approved by Teikyo University Review Board (Approve number 17-069-2) pursuant to the guidelines by the Ministry of Welfare and Labor of the Japanese Government.

Totally, 113 consecutive cases without postmortem degradation (78 males and 35 females) were studied. The causes of deaths (COD) were: 12 heart diseases, 17 other diseases, 28 traumata, 7 asphyxiations, 18 drowning, 4 burning, 10 carbon monoxide intoxications, 5 drug intoxications, one heat shock, one other external cause, and 10 unknown causes. The 12 heart diseases consisted of ischemic heart disease, congestive cardiomyopathy, arrhythmia, and cardiomegaly.

#### Morphometrical study for fibrous tissues

The hearts were fixed in 16% formalin for 1 week, and horizontal sections of the ventricles were embedded in paraffin, and sectioned in 3 μm thickness. Images from sections stained with Masson Trichrome were captured using Olympus microscope attached to DP73 camera, and processed by cellSense Dimension software (Olympus, Tokyo, Japan). The percentage of the area of the blue-stained interstitial fibrous tissue in the middle of the myocardium of the left anterior ventricle (about one-fifth of the horizontal section of the entire left ventricle) was calculated. The massive fibrous tissues from the old myocardial infarction due to the proximal coronary artery occlusion were excluded, since the study focused on the microvascular ischemia and myocardial remodeling. The measurement was carried out by one person who had no knowledge of the case data.

#### Immunohistochemistry of CML and Receptor of AGE (RAGE)

Frozen sections (6 μm) from the left ventricular muscles from anterior, lateral, posterior, and septal areas were made, and stained with the anti-CML antibody (TransGenic, Japan, 1:400 dilution) by the BOND RX automated immunostainer (Leica, Germany) using BOND polymer refine detection system [10].

**Fig. 1:**
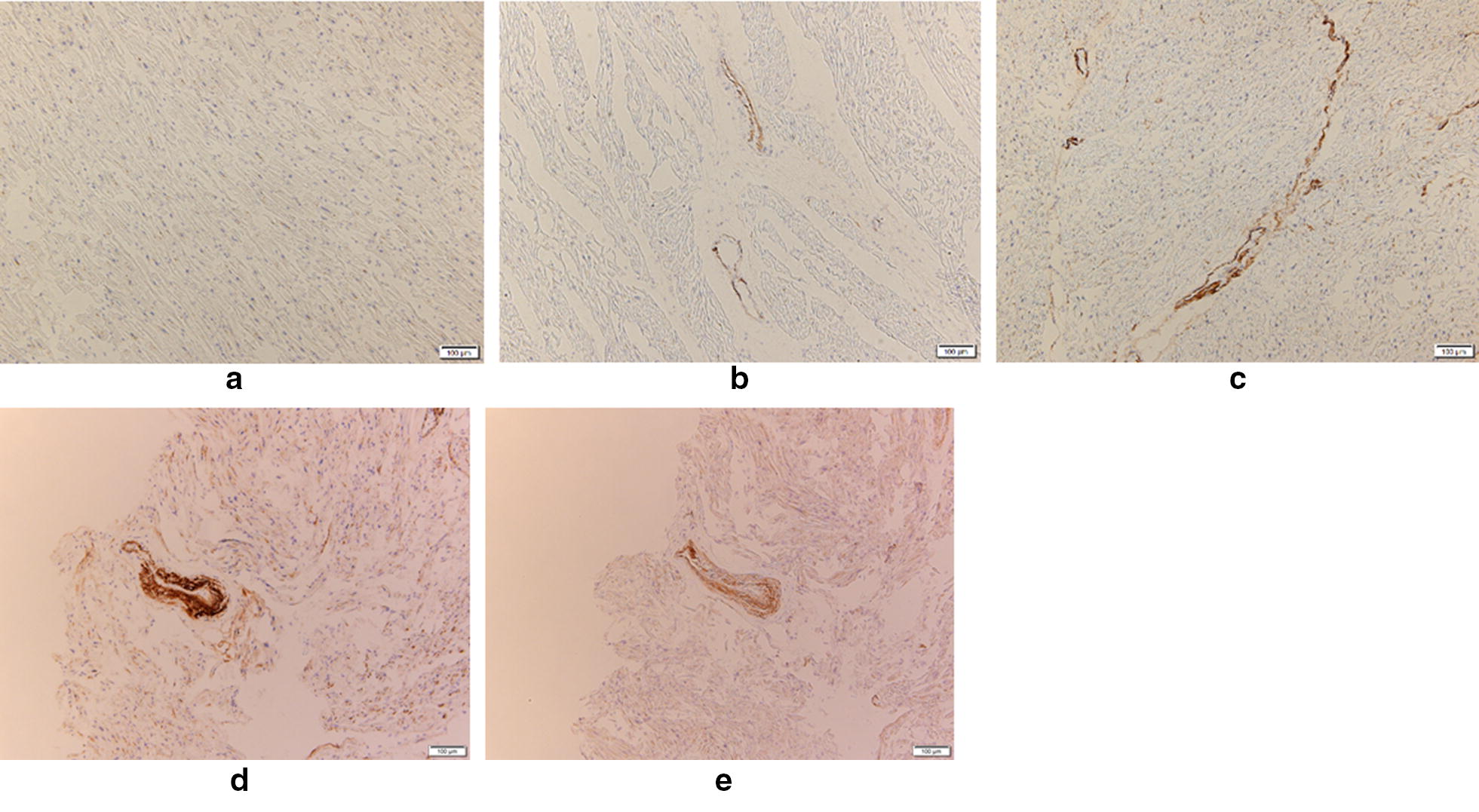
The representative immunohistochemistry for CML and RAGE. The **a**–**c** are representative immunohistochemistry of CML for grade 0 (negative), 1 (weak to middle intensity), and 2 (high intensity), respectively. Note that CML is positive for the endothelial cells and intima of peripheral coronary arteries. The **d** and **e** represent the staining with CML (**d**) and RAGE (**e**) on the sequential sections. Note that CML co-localizes with RAGE. The scale bars in the right bottom show 100 micrometers

The immunohistochemistry of CML in the endothelial cells and intima of the small arteries in the interstitium of the ventricular muscle tissue were classified into three grades: 2 (strong immunostaining), 1 (weak to middle immunostaining), and 0 (negative immunostaining). The representative figures for the CML grades are shown in Fig. 1. The intensity of the staining were uniform in the sections, and the evaluation was carried out by two persons independently without the knowledge of the case data. CML grades were determined for each case using the sections throughout the left ventricles as above.

In order to demonstrate the relevance of the CML, its receptor of AGE (RAGE) was immunohistochemically stained on the sequential frozen sections with the antibody against RAGE (rabbit polyclonal, ab3611, Abcam, UK, 1:500 dilution), and with the anti-CML antibody, using the BOND RX automated immunostainer as above.

#### HbA1c and NT-proBNP measurement

Serum hemoglobin A1c (HbA1c) and NT-proBNP levels were measured consecutively where serum samples were available in cases within postmortem intervals of 3 days (n = 64 for HbA1c, and n = 68 for NT-proBNP), in which 5 heart disease cases were included. Serum HbA1c was measured by Latex agglutination method using Rapidia Auto kit (Fuji Lebio, Tokyo, Japan), and serum NT-proBNP was measured by ECLIA (electro chemiluminescence immunoassay) using Eclusis NT-proBNP II (Rosch Diagnostics, Tokyo, Japan).

### Statistics

The data were expressed as mean ± SEM, and analyzed using ANOVA and Holm-Sidak’s multiple comparisons test in Prism v7.0 software (GraphPad, La Jolla, USA). For the correlation between the history of diseases and the CML immunohistochemistry, Chi square test was used using Prism software. The *p* value less than 0.05 was considered statistically significant. For NT-proBNP and the percentage of cardiac fibrosis, the ROUT (Q = 0.1%) test in Prism software was used to exclude only definite outliers.

### Results

#### The CML and RAGE immunoreactivity in cardiac tissues

**Fig. 2:**
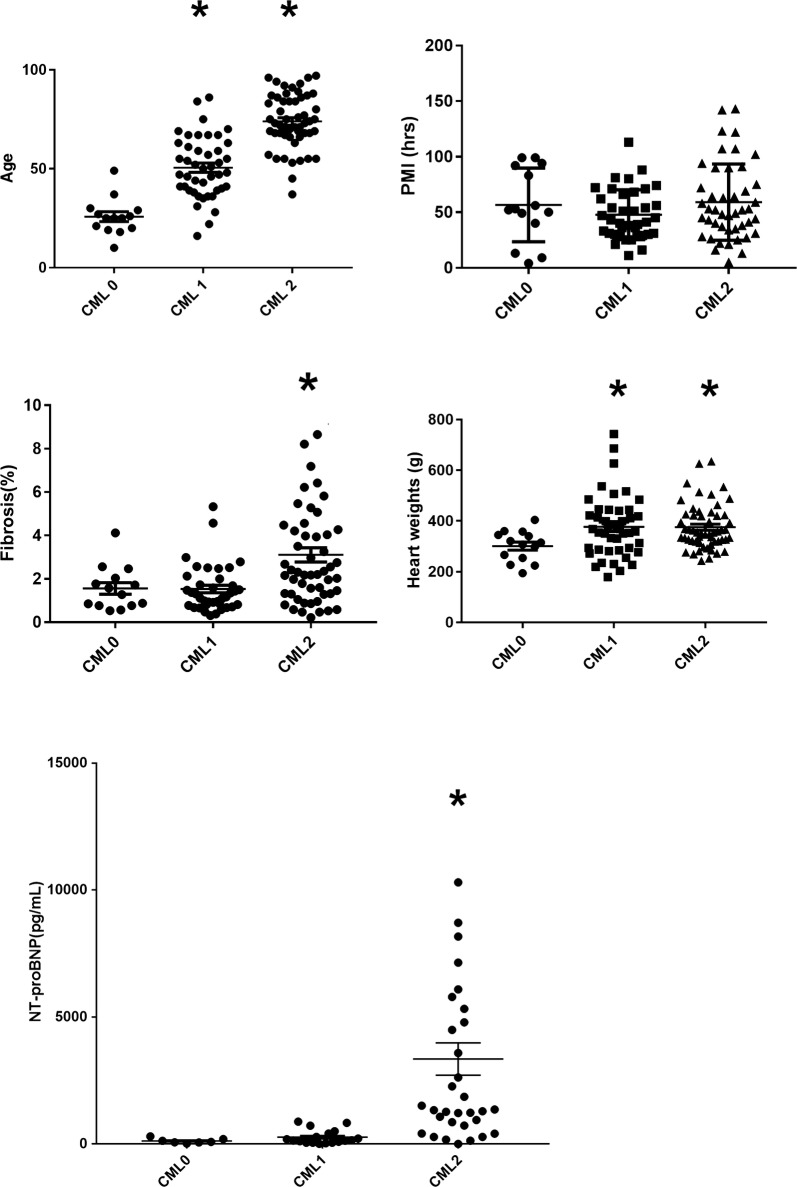
The CML immunohistochemistry in relation to the age, postmortem intervals (PMI), the percentage of myocardial fibrosis, heart weights, and NT-proBNP. The bars show the mean ± SEM. The asterisks in the age show the statistically significant difference from CML 0. The asterisk in the percentage of myocardial fibrosis shows the statistically significant increase in CML grade 2, compared with CML grade 0 and 1. Asterisks in heart weights show the statistically significant increase in CML grade 1 and 2, compared with CML grade 0. The asterisk in NT-proBNP values shows the statistically significant increase in CML grade 2, compared with CML grade 0 and 1

The CML was immunohistochemically positive in the endothelial cells and intima of the small coronary arteries in the interstitium of ventricular muscles (Fig. 1). Anterior, lateral and posterior areas showed similar results. There was no correlation between the CML immunoreactivity and postmortem intervals (PMI, Fig. 2).

The relevance of CML is supported by the immunohistochemical colocalization of RAGE in the endothelial cells and intima of the interstitial arteries (Fig. 1d, e).

#### Correlation between the cardiac CML immunoreactivity and age

We studied the correlation between the age and the immunohistochemical CML-positivity in the small arteries in the interstitium of the ventricular muscles. The means of ages of the CML-positive cases were statistically significantly higher than of the CML-negative cases (25.8 ± 2.5 years for CML 0 cases, 50.6 ± 2.3 years for CML 1 cases, and 73.9 ± 1.9 years for CML 2 cases, Fig. 2).

#### Correlation between the cardiac CML immunoreactivity and heart conditions including the degree of fibrosis and serum NT-proBNP

The CML immunoreactivity (grade 2) had a statistically significantly higher NT-proBNP values in autopsy blood samples than grade 0 and 1 (122 ± 37 pg/mL for CML 0 cases, 268 ± 67 pg/mL for CML 1 cases, and 3346 ± 634 pg/mL for CML2 cases, Fig. 2). Our results also showed that heart weights for CML 1 and 2 showed statistically significantly higher than for CML 0 (301 ± 16 g for CML 0 cases, 377 ± 19 g for CML 1 cases, and 376 ± 12 g for CML 2 cases, Fig. 2).

Interestingly, the degree of interstitial myocardial fibrosis in histology had the statistically significant difference between CML 0 and 2, and CML 1 and 2 (1.57 ± 0.27% for CML 0 cases, 1.53 ± 0.18% for CML1 cases, and 3.11 ± 0.33% for CML 2 cases, Fig. 2).

**Fig. 3:**
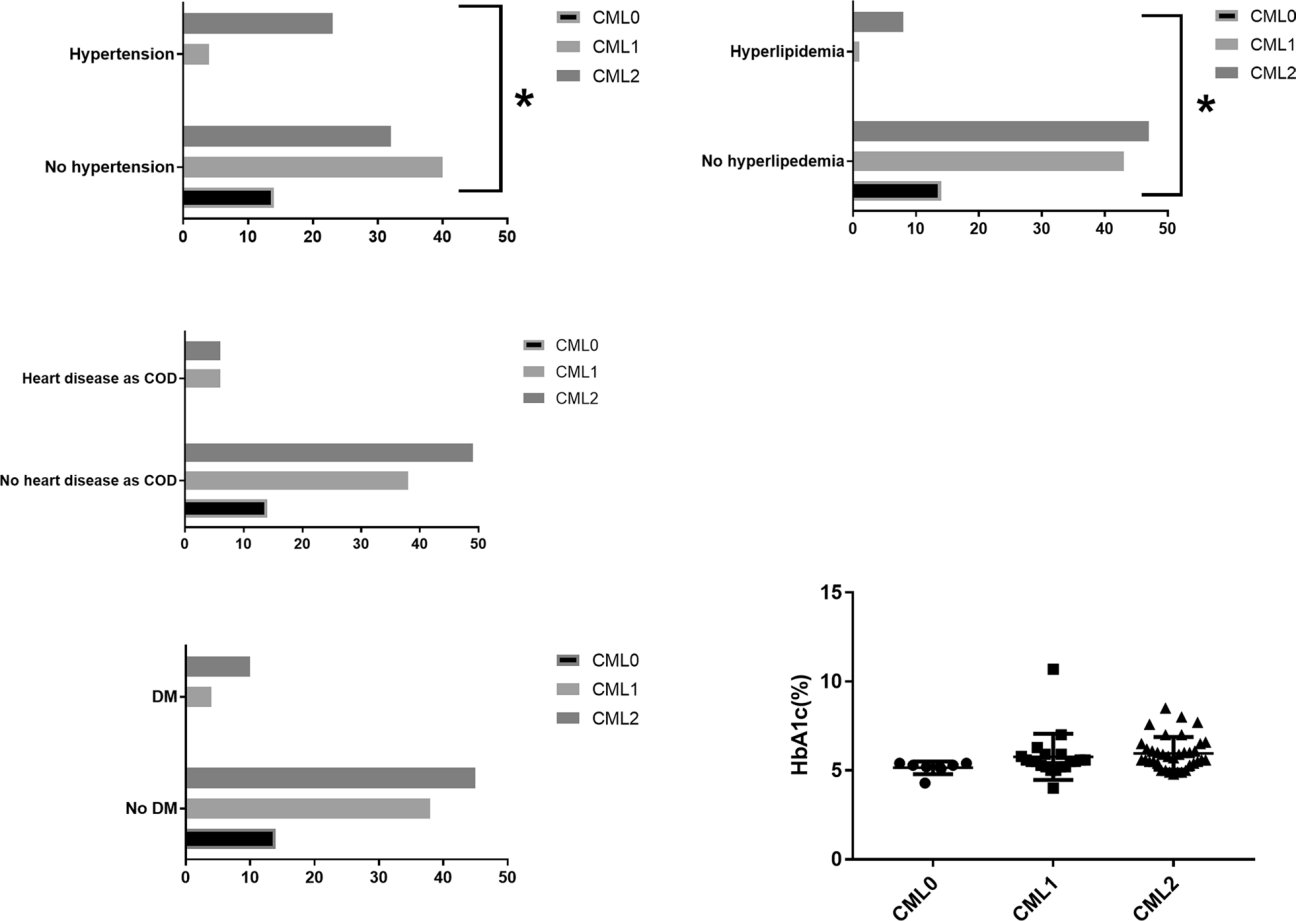
The CML immunohistochemistry in relation to hypertension, hyperlipidemia, heart disease as the cause of death (COD), diabetes mellitus, and HbA1c. The right axis shows the number of cases. Asterisks show the statistically significant difference in disease cases, compared with non-disease cases by Chi square test. The histories of hypertension and hyperlipidemia showed the statistically significant difference in CML immunohistochemistry grades. The heart disease (ischemic heart disease, congestive heart failure, or cardiomegaly) as COD showed no statistically significant difference. The history of diabetes mellitus (DM) and HbA1c showed no statistically significant difference in CML immunohistochemistry grades

Although all cases with heart diseases (ischemic heart disease, chronic congestive heart failure, or hypertensive cardiomegaly) as COD showed the positive CML immunoreactivity, no statistically significant correlation was detected between the heart disease as COD and the CML immunoreactivity (Chi square test, Fig. 3). On the other hand, the history of hypertension had the statistically significantly positive correlation with the CML immunoreactivity (Chi square test, Fig. 3).

#### The cardiac CML immunoreactivity and the history of hyperlipidemia and diabetes mellitus

The history of hyperlipidemia showed the statistically significant correlation with the CML immunoreactivity (Chi square test, Fig. 3). On the other hand, there was no statistically significant correlation between the CML immunoreactivity and the history of diabetes mellitus (Chi square test, Fig. 3) or serum Hb A1c (Chi square test, Fig. 3).

### Discussion

Our results clearly indicate that the CML is immunohistochemically positive in the endothelial cells and intima of the small coronary arteries in the interstitium of ventricular muscles (Fig. 1). Baidoshvili et al. indicated that CML present on activated endothelium in small intramyocardial blood vessels might reflect an increased risk for AMI [3]. CML has received considerable interest, because it can act as a ligand for the RAGE, and it has been associated with increased oxidative stress [11]. Our observation shows the presence of CML in the endothelial cells and the intima of the small arteries in the interstitium of ventricular muscles. Our results support the previous results by Schleicher et al., which showed that the inner elastic lamina of the subcutis arteries was positive for CML-staining [12]. Our result also confirms the finding that the cardiac vasculature has the age-related CML accumulation (Fig. 2) shown by Hu et al. [4]. The significance of CML is supported by the immunohistochemical colocalization of RAGE in the endothelial cells and intima of the arteries (Fig. 1) [13]. Our result shows a novel correlation between the CML immunoreactivity and NT-proBNP, which is a marker for heart failure (Fig. 2). NT-proBNP emerged as a potential biomarker of clinical interest in heart failure management. NT-proBNP is related to heart failure severity and to clinical status. NT-proBNP is strongly associated with prognosis across the whole spectrum of heart failure patients [14]. Our results also show that heart weights have the positive correlations with the CML immunoreactivity (Fig. 2). These results indicate the significance of the CML accumulation in the development of heart failure.

There was the statistically significant difference in the degree of fibrosis in the cardiac tissue between CML 0 and 1, and CML 1 and 2 (Fig. 2). Myocardial ischemia is caused by coronary microvascular dysfunction, which causes myocardial ischemia localized in small, patchy diffuse areas of myocardium [6]. Diffuse myocardial fibrosis is observed as myocardial remodeling in congestive heart failure [7]. Our results on myocardial fibrosis indicate that the CML accumulation in the coronary microvasculature help progress the myocardial fibrosis, which would offer the evaluation of myocardial ischemia and heart failure.

Our results failed to show the statistically significant difference in the degree of the CML immunoreactivity in the coronary microvasculature between cases with heart diseases as COD and other cases (Fig. 3). This may be explained by the fact that the CML accumulation is not the sole cause of heart diseases including ischemic heart disease, hypertensive cardiomegaly, and chronic congestive heart failure. On the other hand, since all cases with heart diseases as COD showed the CML immunoreactivity, further study is warranted for the involvement of CML accumulation in the progression of heart diseases.

The histories of hyperlipidemia and hypertension show the statistically significant correlation with the positive CML immunoreactivity, whereas diabetes mellitus did not (Fig. 3). This indicates that the CML accumulation may occur in the presence of hyperlipidemia or hypertension, independent of the presence of diabetes mellitus.

### Conclusion

Our results show the CML immunoreactivity in the endothelial cells and the intima of the small arteries in the interstitium of the human heart muscles. The CML immunoreactivity increased in intensity with the age. The CML immunoreactivity also shows the positive correlation with the presence of high serum NT-proBNP, which is a novel indication of the CML involvement in heart failure.　Interstitial fibrosis in the cardiac tissue showed the significant increase in the group with the high CML immunoreactivity. Heart weights, the histories of hypertension, and hyperlipidemia also show the positive correlation with the CML immunoreactivity.　Our results warrant the further study for the role of CML deposition in the cardiac microvasculature for the evaluation of ischemic heart disease.

## Limitations

Correlations between CML immunoreactivity and various factors do not directly prove that CML is the cause for the disease states.

## Data Availability

All data generated or analyzed during this study are included in this published article.

## Abbreviations

CML: N^ε^-(carboxymethyl)lysine
AGE: Advanced glycation end products
RAGE: Receptor of AGE
NT-proBNP: N-terminal-pro basic natriuretic peptide
COD: Cause of death
PMI: Postmortem interval
